# Investigation of Gallium(III) Complexes with Thiouracil Derivatives: Effects of pH on Coordination and Stability

**DOI:** 10.3390/ijms252312869

**Published:** 2024-11-29

**Authors:** Monika Skrobanska, Michał Zabiszak, Anita M. Grześkiewicz, Malgorzata T. Kaczmarek, Renata Jastrzab

**Affiliations:** Faculty of Chemistry, Adam Mickiewicz University, Uniwersytetu Poznanskiego 8, 61-614 Poznan, Poland; zabiszakm@amu.edu.pl (M.Z.); anita.grzeskiewicz@amu.edu.pl (A.M.G.); gosiat@amu.edu.pl (M.T.K.); renatad@amu.edu.pl (R.J.)

**Keywords:** thiouracil, gallium(III) ions, potentiometric titration, single X-ray

## Abstract

This study explores the formation and properties of new complexes involving gallium(III) and thiouracil derivatives—2-thiouracil (TU), 6-methyl-2-thiouracil (MTU), 6-propyl-2-thiouracil (PTU), 5-carboxy-2-thiouracil (CTU), and 6-methoxymethyl-2-thiouracil (MMTU). Conducted in aqueous solutions at relatively low concentrations, this research enabled the formation of soluble complexes, identified and described here for the first time. The influence of metal-to-ligand ratios on species distribution and their fluorescence properties was examined through potentiometric titration, alongside visible and fluorescence spectroscopy. Stability constants were determined, revealing that coordination mode and complex stability are pH-dependent, and nitrogen, sulfur, and oxygen atoms are involved in higher pH coordination. Additionally, the structure of the ligand 6-methoxymethyl-2-thiouracil was characterized. The findings suggest that these complexes hold potential for future biomedical applications, particularly as antibacterial and anticancer agents, warranting further studies under physiological conditions.

## 1. Introduction

Metals play an indispensable role in life—especially metal ions, which significantly influence the biochemistry processes and functions of the living organism. The applications of gallium ions are well known in the literature [1] that cover areas ranging from antitumor drugs [2,3,4] to antibiotics [5,6,7] and also radiography [8,9,10,11]. Specifically, gallium compounds have demonstrated promising antibacterial and anticancer activities. However, simple gallium salts exhibit significant imperfections in in vivo studies. For instance, gallium nitrate causes nephrotoxicity and optic nerve neuropathy, while gallium chloride has negligible bioavailability. To address these challenges, complex gallium compounds, particularly those with therapeutic ligands, have emerged as a promising alternative approach. Most of the gallium(III) antitumoral [12,13,14] or antibiotic [15,16] activity comes from its similarity to iron(III) [12], which allows Ga^3+^ ions to enter iron metabolic pathways and break them.

Recent studies in the literature highlight gallium complex compounds with flavonoids [17] or thiosemicarbazones [1], as well as nanocrystalline structures with tetraphenyl porphyrin [18]. The scant number of literature reports on studies of the complexation reactions of gallium ions with thio-ligands has led to research involving compounds such as uracil and its derivatives, which have been successfully used as therapeutics in cancer treatment, as well as in the treatment of diabetes and AIDS.

The thio-derivatives of uracil are considered privileged structures in drug discovery with a wide array of biological activities. Their versatility arises from their ability to change properties by complexing various molecules or metal ions in nitrogen, carbon, oxygen, and sulfur atoms [19].

The stability of metal complexes at different pH levels is essential in determining their biological activity. In living organisms, metal ions frequently interact with various ligands, such as proteins, enzymes, and other biomolecules, to form complexes. Alterations in the coordination environment, such as pH shifts, ligand substitution, or the detachment of the metal from the complex, can greatly affect the nature of their biological activity. Therefore, comprehending and regulating the stability of metal complexes are crucial for understanding their roles in biological systems and advancing applications in areas like medicine.

**Figure 1:**
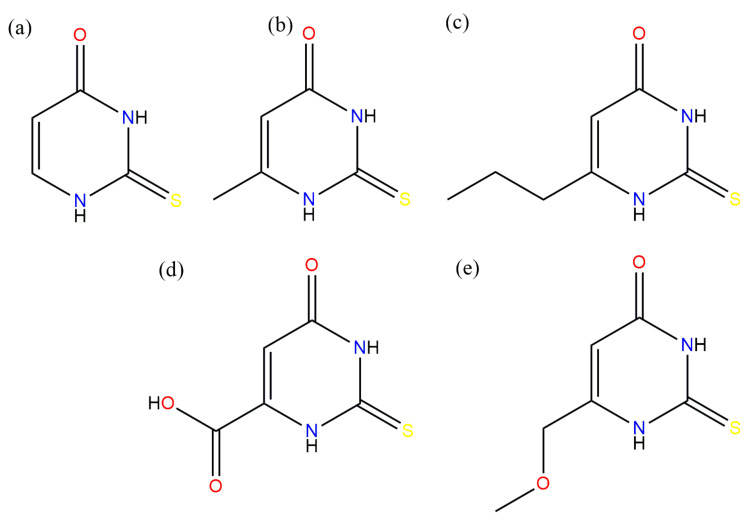
Chosen thiouracil ligands: (**a**) 2-thiouracil, (**b**) 6-methyl-2-thiouracil, (**c**) 6-propyl-2-thiouracil, (**d**) 5-carboxy-2-thiouracil, (**e**) 6-methoxymethyl-2-thiouracil.

This work focused on the potentiometric titration of several thiouracil derivatives (Figure 1) and the characterization of novel coordination compounds with gallium ions.

It is worth highlighting that 6-methoxymethyl-2-thiouracil (MMTU) and 6-propyl-2-thiouracil (PTU, anti-thyroid drug) can certainly be regarded as structural analogs; the only difference is the presence of an oxygen atom (MMTU) or methylene group (PTU) at position 2 in the three-atom sidechain. This small difference significantly affects the packing in the molecular crystals of both compounds.

Both thioamides, MMTU and PTU, form many multicomponent crystals—solvates, co-crystals, or salts—mainly due to their potential as both hydrogen bond donors and/or acceptors [20,21,22,23]. Strangely, despite a quite significant number of compounds including MMTU deposited in the Cambridge Structural Database (CSD) [24], there are no data regarding pure MMTU. Therefore, because we were planning to use both thioamides in attempts to prepare the complexes, we decided to try to determine the structure of MMTU without solvents or other co-formers. These data allowed us to look for similarities or differences in the intermolecular bond formation of both thioamides.

## 2. Results and Discussion

### 2.1. Potentiometric Titration Luminescence Spectroscopy

2-thiouracil (TU) (Figure 1a), 6-methyl-2-thiouracil (MTU) (Figure 1b), and 6-propyl-2-thiouracil (PTU) have three potential coordination sites at (i) the nitrogen atom, (ii) the sulfur atom, and (iii) the oxygen atom. 5-carboxy-2-thiouracil (CTU) (Figure 1d) and 6-methoxymethyl-2-thiouracil (MMTU) (Figure 1e) possess an additional coordination site at the oxygen atom of the carboxyl group. The first step of the study involved determining the protonation constant of the ligands.

TU log*K* = 6.95, MTU log*K* = 7.71, PTU log*K* = 7.69, CTU log*K* = 4.01, and MMTU log*K* = 6.58. Moreover, the constants for the hydrolysis of gallium ions were calculated; log*β* for Ga(OH) is −4.25, and for Ga(OH)_2_, it is −13.54.

The value of the protonation constant determined for the ligands by computer analysis of the titration data corresponds to the protonation that occurs in the nitrogen atom N(3).

Due to the problem with the solubility of thiouracil derivatives, potentiometric titration was performed at a relatively low concentration of 0.5 mmol.

Computer analysis of potentiometric data across all systems suggests the formation of hydroxido complexes in both 1:1 and 1:2 ratios. The resulting complex forms, stability constants, and equilibrium constants for the complex formation reactions observed in the studied systems are summarized in Table 1.

**Figure 2:**
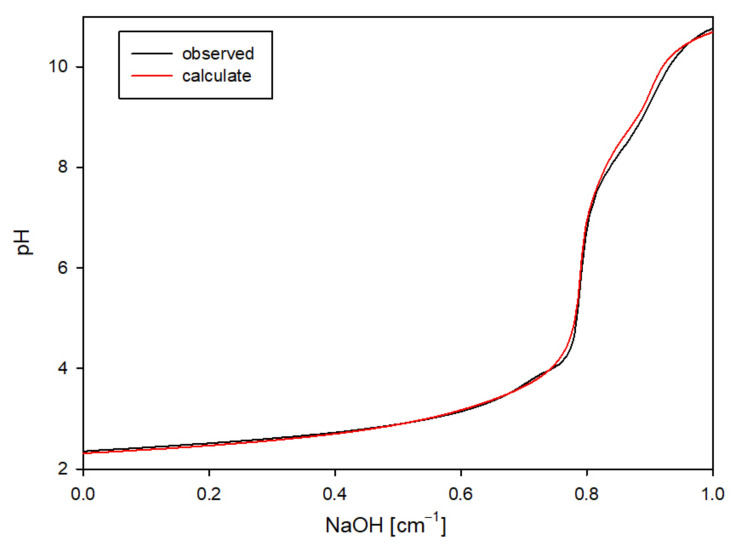
An example comparison of the experimental curve with the theoretical one for the MTU ligand.

To verify the accuracy of the selected complex set, theoretical curves were generated using the HYSS program, incorporating the stability constants of the formed complexes in the calculations. The strong agreement between the theoretical and experimental curves validated that the proposed complexes were indeed formed in the system (Figure 2) [25].

#### 2.1.1. Ga/TU System

**Figure 3:**
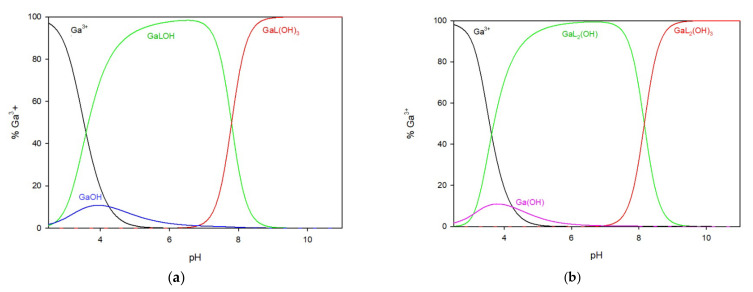
The distribution of complex species in the Ga/TU system, (**a**) 1:1 ratio, (**b**) 1:2 ratio.

In the system of 2-thiouracil with gallium ions, a 1:1 metal/ligand ratio (Figure 3a) mixture of complexes was formed in the whole pH range. The GaLOH complex is present from the beginning of the titration process and, within the pH range of 5.0 to 7.5, binds more than 95% of the gallium ions. The second hydroxy complex, GaL(OH)_3_, forms at pH = 7.0 and reaches its maximum at pH 8.0, binding 100% of the gallium ions in the solution, and remaining until the end of the study.

In the system with a metal-to-ligand ratio of 1:2 (Figure 3b), complexes are sequentially formed by adding ligand molecules to the inner coordination sphere, as shown in Table 1. The complex GaL_2_(OH) is present in the system at a low pH of around 3.0 but disappears at approximately pH 9.0. At pH 9.0, the predominant complex is GaL_2_(OH)_3_, which represents almost 100% of the gallium ions in the solution.

#### 2.1.2. Ga/MTU System 

**Figure 4:**
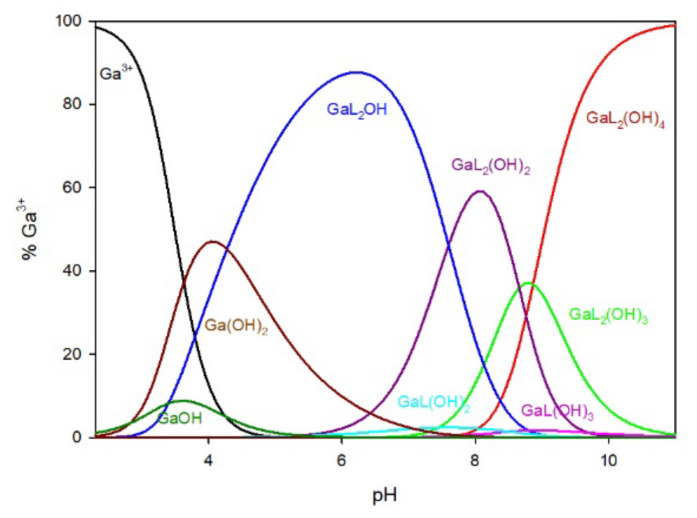
The distribution of complex species in the Ga/MTU system.

The distribution of complex species formed in the Ga/MTU (L) system is shown in Figure 4. The complexation reaction begins at pH 3.0 with the formation of the Ga(MTU)_2_OH complex, which reaches its maximum at pH 6.0, where approximately 85% of gallium ions are bound. Other species in the Ga/MTU system that bind over 40% of the gallium ions have maximums at pH 8.0 (GaL_2_(OH)_2_), pH 8.5 (GaL_2_(OH)_3_), and pH 10 (GaL_2_(OH)_4_).

#### 2.1.3. Ga/PTU System

**Figure 5:**
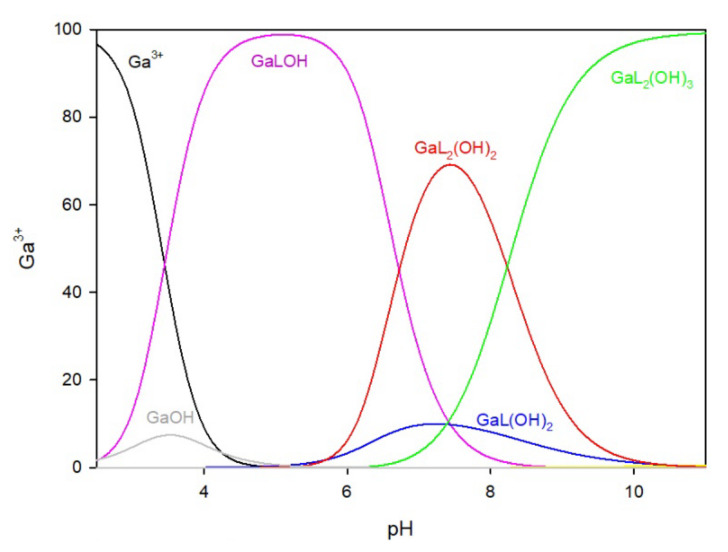
The distribution of complex species in the Ga/PTU system.

For systems with 6-propyl-2-thiouracil in 1:1 and 2:1 ratios (Figure 5), the presence of the GaLOH complex form is observed from the beginning of the titration process, reaching its maximum in the pH range of 4.0–6.0, binding approximately 90% of the gallium ions in the solution. The GaL(OH)_2_ and GaL_2_(OH)_2_ complex forms appear at a pH of around 4.0. The first form reaches its maximum at pH 7.0, binding about 10% of the gallium ions. By contrast, the GaL_2_(OH)_2_ form reaches its maximum at pH 7.5, binding approximately 65% of the gallium ions. The last observed form is GaL_2_(OH)_3_, which forms at pH 6.5 and reaches its maximum at pH 9.5, binding about 90% of the gallium ions.

#### 2.1.4. Ga/CTU System

**Figure 6:**
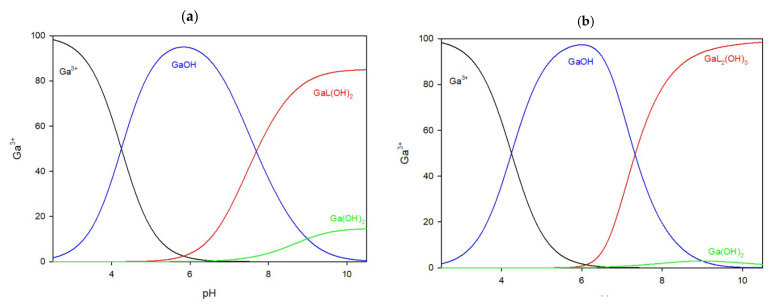
The distribution of complex species in the Ga/CTU system, (**a**) 1:1 ratio, (**b**) 1:2 ratio.

In the system with the 5-carboxy-2-thiouracil ligand, the complexation of gallium ions begins at pH 5.5 in both the 1:1 and 2:1 ratios. Before this pH, only free gallium ions and gallium hydroxide are detected. In the 1:1 system (Figure 6a), the only complex, GaL(OH)_2_, reaches its peak at pH 8.5, binding 85% of the gallium ions in the solution. When the ligand is in excess (Figure 6b), the GaL_2_(OH)_3_ complex forms, peaking at pH 8.5, binding approximately 75% of the gallium ions.

#### 2.1.5. MMTU/Ga System

**Figure 7:**
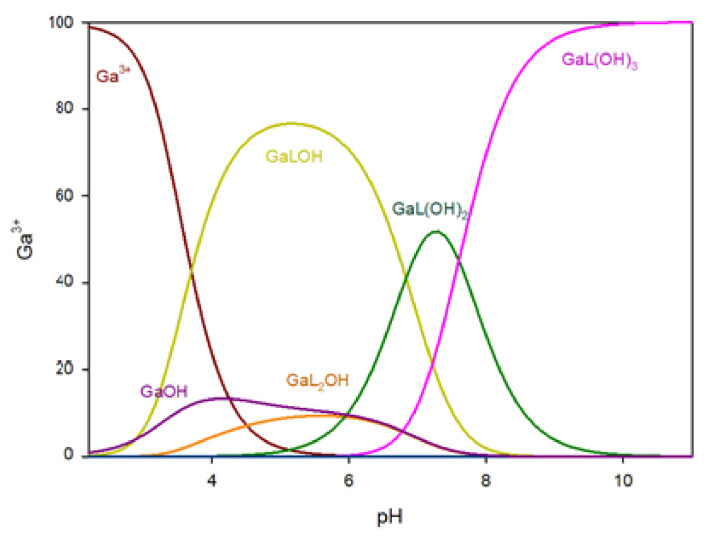
The distribution of complex species in the Ga/MMTU system.

The distribution of complex species in the Ga/MMTU (L) system is illustrated in Figure 7. Complexation begins at pH 3.0 with the formation of the Ga(MMTU)OH complex, which peaks at pH 5.0, binding about 75% of the gallium ions. In the Ga/MMTU system, the GaL(OH)_2_ complex binds around 55% of the gallium ions at pH 7.5, while at pH levels greater than 8.5, only the GaL(OH)_3_ complex is present.

### 2.2. Spectroscopic Studies

#### 2.2.1. UV-Vis

Spectroscopic methods were employed to study the formation of complex compounds. UV-Vis measurements were conducted at pH levels where the highest concentration of each specific form was observed, and these pH values were chosen based on distribution curves.

**Figure 8:**
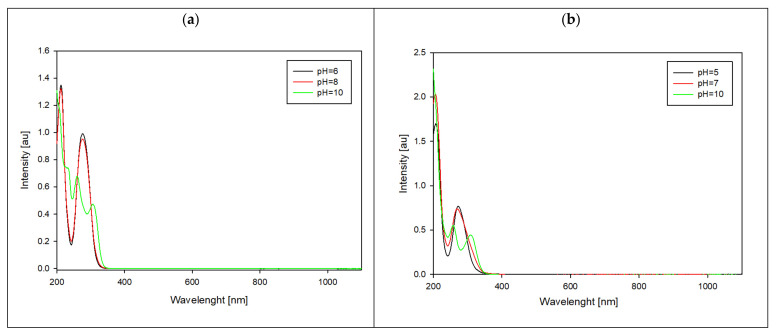
UV-Vis spectra in different pH values for systems: (**a**) Ga/MTU and (**b**) Ga/MMTU.

The shifts in the absorbance toward longer wavelengths indicate changes within all of the complexes’ internal coordination spheres. Additionally, a new peak emerges at higher pH levels (Figure 8). According to the literature data [26], at a lower pH, the complexation process primarily involves a nitrogen atom (N3). On the contrary, at higher pH levels (above 9), coordination occurs through both oxygen and sulfur atoms.

#### 2.2.2. Luminescence Spectroscopy 

**Figure 9:**
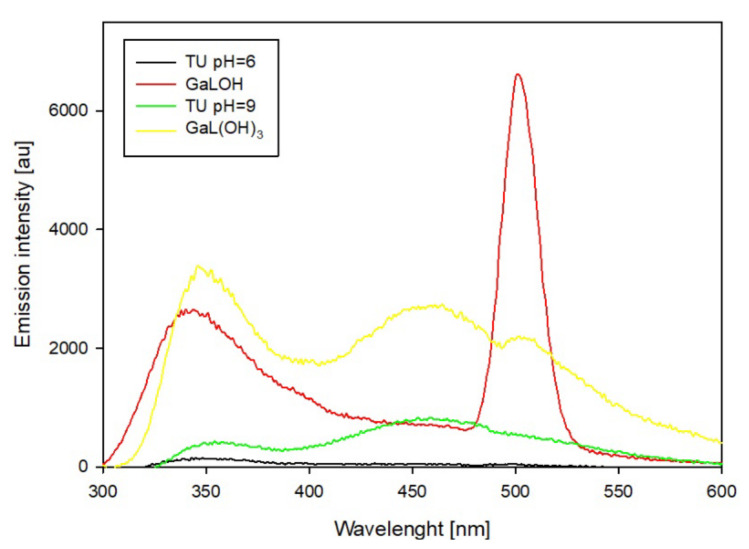
Emission spectra of the Ga/TU system.

Measurement conditions for the luminescence studies were selected on the basis of distribution diagrams of the systems studied and UV-vis spectroscopy studies of the ligands. The excitation wavelength corresponds to the absorption maximum of the ligand, and the pH conditions correspond to the pH values of the dominant complex forms. Changes in the emission intensity of the studied thiouracil derivatives and their complexes are observed in the range of 280–600 nm, depending on the chemical modifications of the functional groups. Changing the pH conditions affects the protonation changes in the functional groups, which changes the electron and coordination properties of the compound. The thiouracil derivative itself may have relatively weak luminescence emission as a result of the competition of nonradiative relaxation processes (e.g., by vibrations of the molecule). The presence of gallium ions in the system with 2-thiouracil increases the emission intensity as a result of the formation of coordination bonds of the ligand’s donor groups. The largest change is observed at 500 nm, which may be due to the attachment of another donor atom (Figure 9). The disappearance of emission at pH 9.0 may indicate changes in the electron structure of the complex (change in the internal coordination sphere of the central atom). Changes in the environment can affect the relaxation of excited states so that radiative emission processes become less efficient.

**Figure 10:**
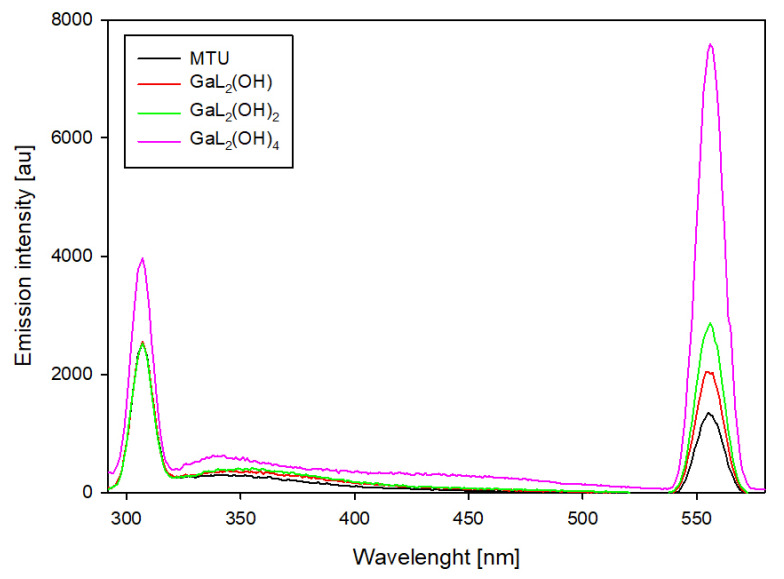
Emission spectra of the Ga/MTU system.

**Figure 11:**
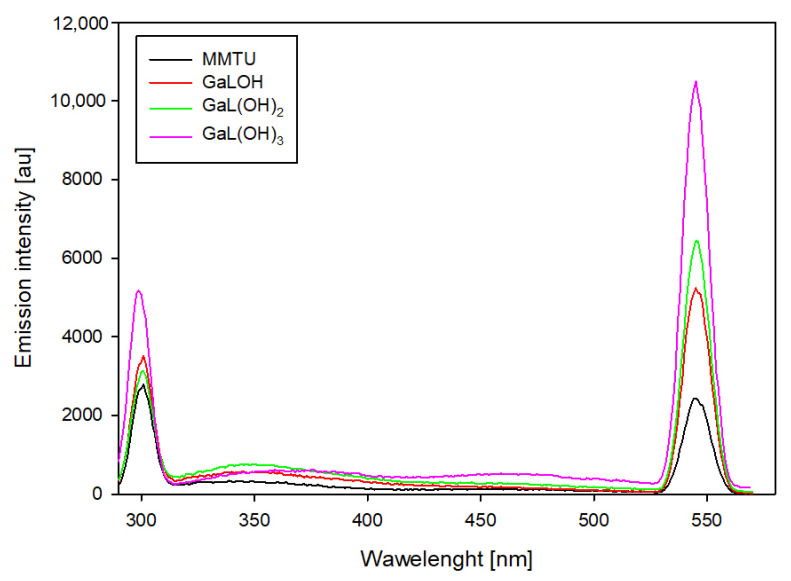
Emission spectra of the Ga/MMTU system.

For binary systems containing 6-methyl-2-thiouracil and 6-methoxymethyl-2-thiouracil, similar changes in emission intensity were observed (Figure 10 and Figure 11). For these systems, the highest emission intensity is observed at 307 nm and 555 nm. The emission intensity at the indicated wavelengths for uncomplexed ligands as the pH increases is similar. The presence of gallium ions in the studied system results in an increase in emission at 555 nm for each of the dominant complexes. Notable is the fact that the higher the pH value, the higher the emission increase. Furthermore, for complexes that dominate above pH 10.0, an increase in emission is also observed at 307 nm.

**Figure 12:**
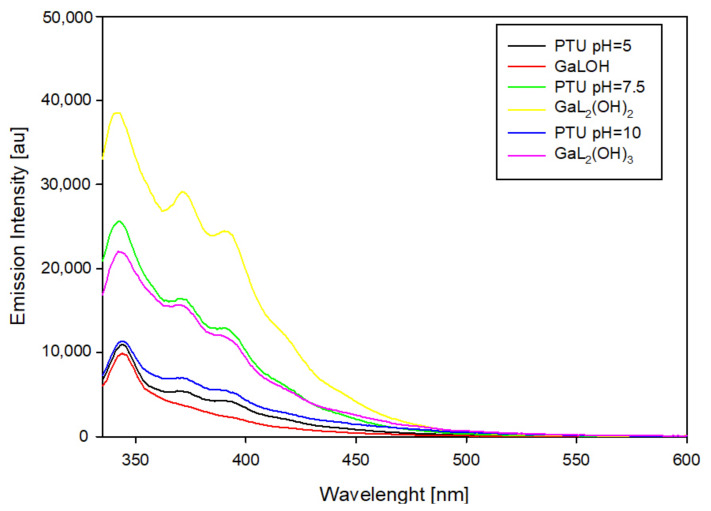
Emission spectra of the Ga/PTU system.

The resulting spectra for systems containing 6-propyl-2-thiouracil (Figure 12) show emission in the range of 335 to 500 nm. A similar emission intensity was found for the dominant complex at pH 5.0 relative to that of the uncomplexed ligand. On the contrary, an increase in pH results in a significant increase in emission, which is observed for the dominant coordination compound at pH 7.5. In contrast to the previously described systems, a further increase in pH results in a gradual extinction of luminescence.

**Figure 13:**
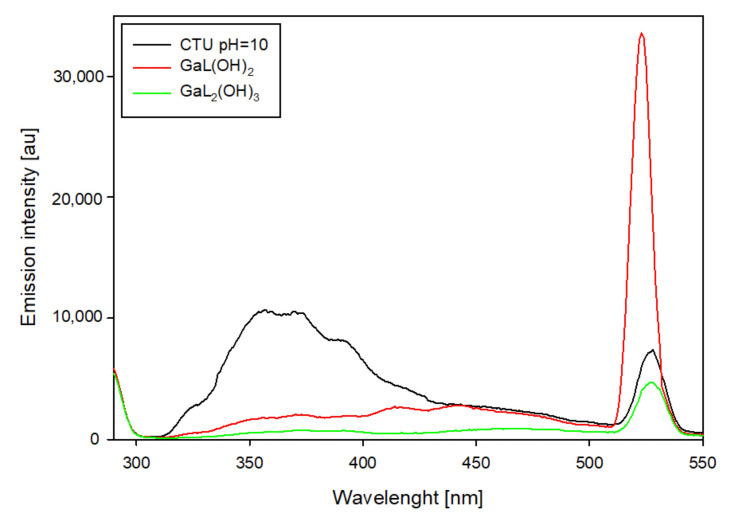
Emission spectra of the Ga/CTU system at pH = 10.0.

In the system containing 5-carboxy-2-thiouracil, changes in emission intensity were also observed (Figure 13). Compared to the free ligand, the occurrence of gallium ions caused emission in the 325–400 nm range. In addition, the emission was found to shift from 527 nm (free ligand) to 522 nm with a significant increase in its intensity.

All of the observed changes in the luminescence studies indicate changes in the internal coordination sphere of the central atom in the studied binary systems. These changes suggest that an increase in pH affects the protonation state of functional groups (e.g., nitrogen in a pyrimidine ring or sulfur), which alters the electron and coordination properties of the compound.

### 2.3. Structural Studies

**Figure 14:**
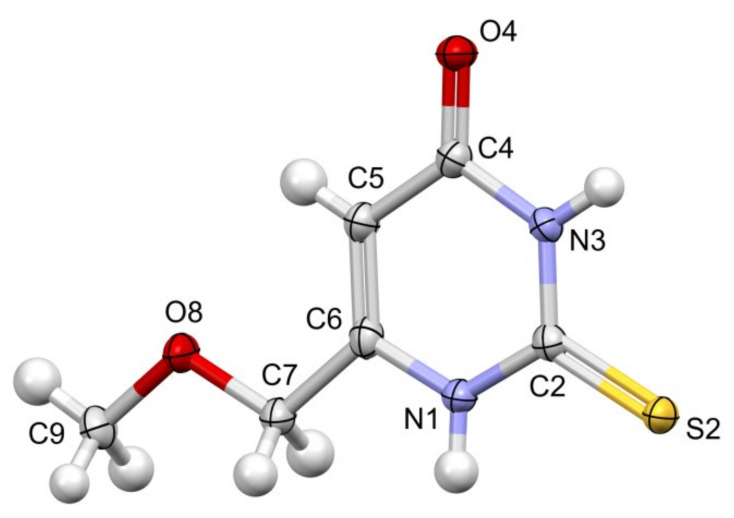
Perspective view of the MMTU molecule, with the numbering scheme; the ellipsoids are drawn at the 50% probability level, and hydrogen atoms are shown as spheres of arbitrary radii. The following colours have been assigned to the atoms: light grey-carbon, white-hydrogen, blue-nitrogen, red-oxygen, yellow-sulfur.

Colorless crystals of MMTU were obtained, suitable for X-ray data collection, using the procedure described in detail in the experimental section. The molecule of MMTU together with the numbering scheme is shown in Figure 14. It crystallizes in the P-1 space group with one molecule in the asymmetric unit, while the crystal structure of PTU contains two such independent molecules. The crystal structure of PTU is quite well known. For the first time, it was published in 1983 [27]; however, for our purposes, we used more accurate data, published in 2011 [28].

At the level of molecular geometry, heterocyclic rings do not differ to a great extent. The most significant difference concerns the orientation of the sidechain (Figure S1 Supplementary Materials) toward the heteroaromatic ring. In both cases, the chains are almost planar (C-C-O(C)-C torsion angles of 174.8(3)° and 178.8(3)° PTU; 177.8(1)°), but in MMTU, the chain is almost coplanar with the aromatic ring (the most deviated atom from the plane of all non-hydrogen atom is 0.0632 (11). Rms deviation of fitted atoms = 0.0314) (dihedral angle (planes defined by atoms: 1st—C9 O8 C7; 2nd—N1 C6 C7) of 7.5(2)°), while in PTU, the corresponding dihedral angles are significantly larger, −25.3°, Figure S1 (Supplementary Materials). In multicomponent crystals, all combinations are present; in 11 out of 15 structures with PTU, the chains are coplanar with the ring. Also, there is an example of MMTU structure, where the sidechain does not lie in the plane. This may lead to the conclusion that the conformation of the sidechain is influenced by the crystal packing.

The presence of strong hydrogen bond donors (NH) and an acceptor (carbonyl oxygen atom; the S atom can also be regarded) results in HB formation. In both compounds, the packing is determined by forming N-H···O and N-H···S ($R_{2}^{2}\left(8\right)$ orange dash line) dimers, which, lying alternately, induce the formation of the $C_{2}^{2}\left(10\right)$ red dash line) chain.

**Figure 15:**
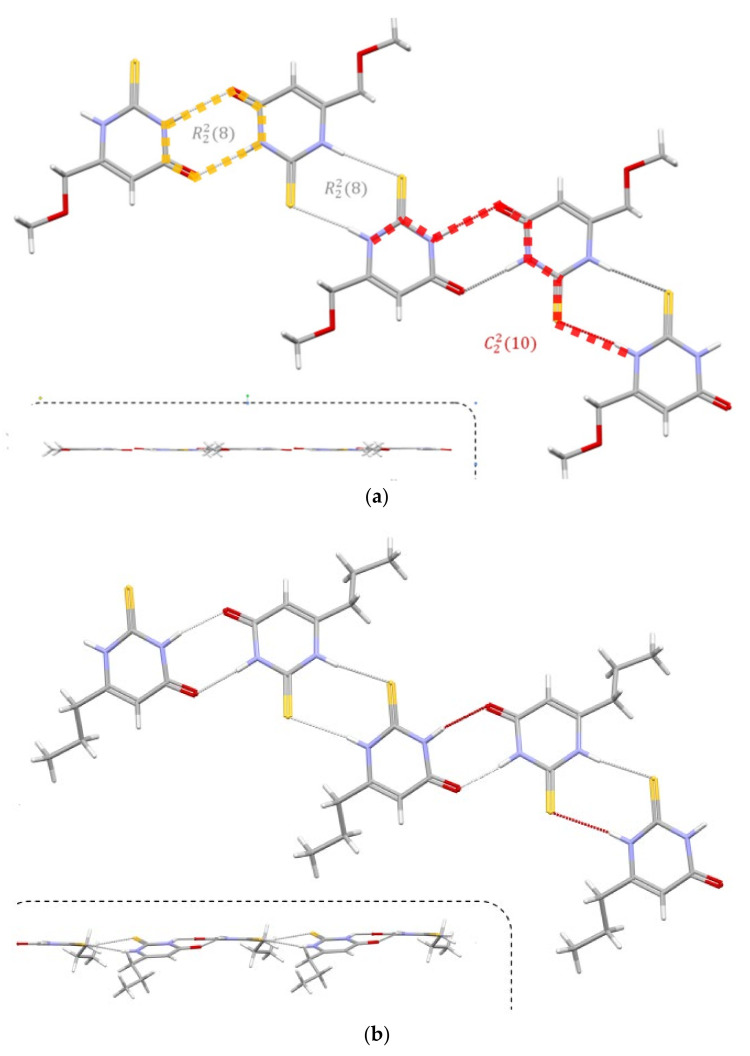
The hydrogen bond pattern in analyzed thioamides. (**a**) MTU, (**b**) PTU. The following colours have been assigned to the atoms: light grey-carbon, white-hydrogen, blue-nitrogen, red-oxygen, yellow-sulfur.

However, in addition to this obvious similarity (Figure 15), the hydrogen-bonded dimers in both thioamides are not perfectly equal. In the MMTU, both N-H···O and N-H···S dimers lie approximately in the plane of the aromatic rings, while in the PTU, the planes defined by the aromatic rings of successive molecules in the chain form an angle of approx~19^°^ Figure 15 (below the dashed line). The geometrical parameters of HB’s arelisted in Table 2. The D···A distances are comparable, but the bonds in the structure of MMTU are closer to linearity.

With respect to this first level of analysis, the supramolecular structures of both PTU and MTU are quite similar, but more substantial differences appear. Thus in MMTU CH_3_ is a donor of the C-H···O hydrogen bond, which forms an $R_{2}^{2}\left(14\right)$ dimer, while in PTU, a similar interaction is not present. MMTU therefore forms a two-dimensional network of hydrogen-bonded molecules that lie almost in one plane (Figure S2, Supplementary Materials).

**Figure 16:**
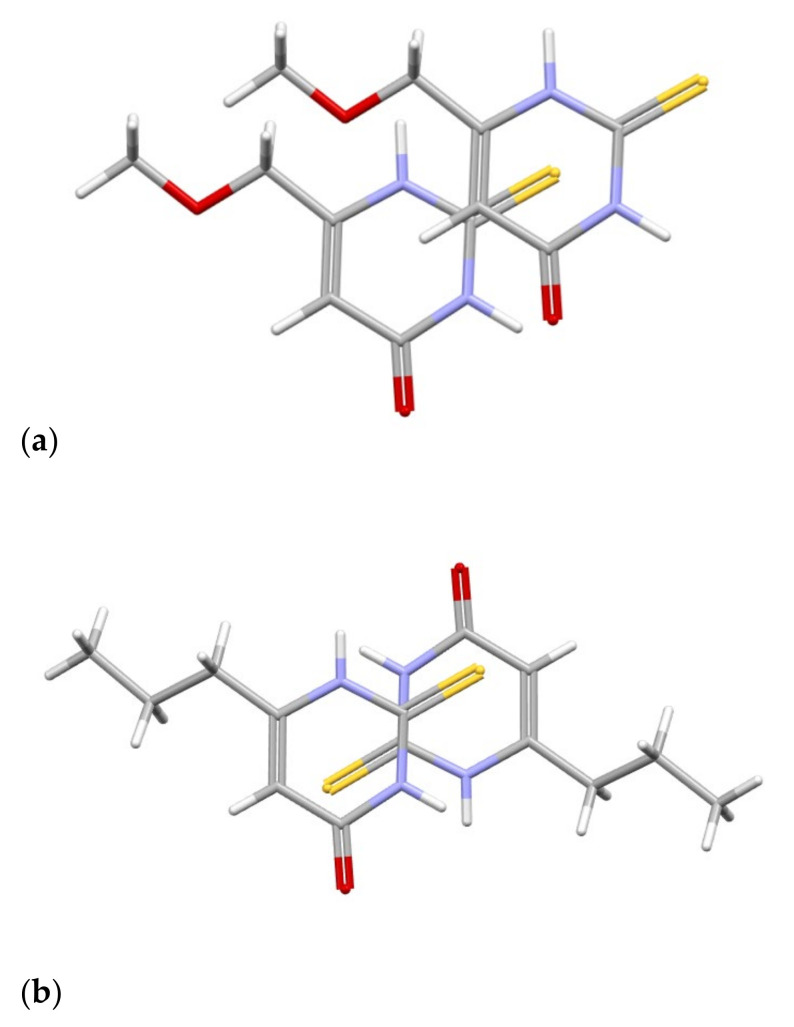
A comparison of S···π interaction in MMTU (**a**) and PTU (**b**). The following colours have been assigned to the atoms: light grey—carbon, white—hydrogen, blue—nitrogen, red—oxygen, yellow—sulfur.

Those layers of MMTU molecules interact with each other by the C-H···O and C-H···S contacts but also by S···π interaction (Figure 16), where the sulfur atom is placed just over (or below) the middle of the aromatic ring. A similar contact, but with some offset, is also present in PTU, which proves its importance in crystal packing. All of these differences result in definitely different packing based on the same synthons (Figure S3, Supplementary Materials).

## 3. Materials and Methods

### 3.1. Materials

All reagents were purchased from Sigma Aldrich (St. Louis, MO, USA) and used without prior purification.

### 3.2. Potentiometric Titration

Potentiometric studies were conducted using a Titrino 702 Metrohm (Zofingen, Switzerland) titration set. The pH meter was calibrated each time before each set of measurements using two buffer solutions at pH = 4.00 and pH = 9.22 at 20 ± 1 °C. Potentiometric titration was carried out at room temperature (20 ± 1 °C) in an inert gas atmosphere (He 5.0) with a constant ionic strength of µ = 0.1 M KNO_3_, using NaOH (0.2023 M) free of CO_2_ as the titrant. All solutions were prepared in ultrapure water, with conductivity below 0.055 µS/cm. The concentration of gallium ions, as well as ligands, was 5.10 × 10^−4^ M. The studies were carried out in metal-to-ligand molar ratios of 1:1 and 1:2 within the pH range of 2.5–11.0. The protonation constants of the ligand and the stability constants of the complexes were determined using computer analysis of potentiometric data with the HYPERQUAD program, and determined ionic products for water was p*K*_w_ =13.78. The calculations were performed using 150–350 points for each set of analysis (ten or more titrations were performed for each system). No precipitate was observed during the titration. Hydrolysis constants of metal ions were also taken into account in the computer analysis of potentiometric data. The iteration procedure allows for the determination of the types (stoichiometry) and thermodynamic stability of the complexes formed in the studied systems. The correctness of the assumed model was verified by analysis of the standard deviations, the convergence of the experimental data with those obtained for the model evaluated by the Hamilton test, and the chi squared test.

### 3.3. Visible Spectroscopy

UV-Vis measurements were performed on an Evolution 300 ThermoFisher spectrophotometer (Thermo Fisher Scientific, Waltham, MA, USA) equipped with a xenon lamp using quartz cells with a 1 cm path length. Measurements were made at room temperature in a wavelength range of 290–1100 nm. The concentration of the ligands and gallium ions were the same as in the potentiometric studies.

### 3.4. Luminescence Spectroscopy Measurements

Samples for luminescence spectroscopy were prepared in ultralight quality water. Water was doubly distilled and purified using a Simplicity Ultrapure Water System (Millipore, Darmstadt, Germany). Luminescence measurements were carried out on a Jasco FP8300 spectrofluorometer with slits set at 5/5 in the range of 290–750 nm at room temperature in a 1 cm × 1 cm quartz cell.

### 3.5. Single Crystal X-Ray Measurement

X-ray diffraction data were collected by the ω-scan technique at 130 K on a four-circle Rigaku SuperNova diffractometer, equipped with Atlas detector and mirror-monochromated CuKα radiation (λ = 1.54178 Å) [30]. The data were corrected for Lorentz–polarization effects, as well as for absorption [31]. Accurate unit-cell parameters were determined by a least-squares fit of the reflections of the highest intensity, which were chosen from the whole experiment. The calculations were carried out mainly within the OLEX2 [32]. The structure was solved with ShelxT [33] and refined with the full-matrix least-squares procedure on F^2^ by SHELXL-2017 [34]. Scattering factors incorporated in SHELXL were used. All non-hydrogen atoms were refined anisotropically, and hydrogen atoms were found in different Fourier maps and freely isotropically refined. Structure representation was prepared with Mercury4.3.0 software [35]. Crystallographic data and refinement details are listed in Table S1. Crystallographic data for the structural analysis were deposited with the Cambridge Crystallographic Data Centre, No. CCDC 2387155.

## 4. Conclusions

This study has explored the complexation of gallium ions with thiouracil derivatives, highlighting the potential of these coordination compounds in therapeutic applications. Our findings indicate that gallium complexes not only mimic the behavior of iron ions but also enhance the biological activity of the ligands involved. By leveraging the unique properties of thiouracil derivatives, we demonstrated their capacity to stabilize gallium ions, potentially overcoming the limitations associated with simple gallium salts.

The results of the thermodynamic and spectroscopic studies showed that thiouracil derivatives involving 2-thiouracil (TU), 6-methyl-2-thiouracil (MTU), 6-propyl-2-thiouracil (PTU), 5-carboxy-2-thiouracil (CTU), and 6-methoxymethyl-2-thiouracil (MMTU) can be formed with gallium(III) ion complexes. The coordination sphere of the gallium ion is complemented by hydroxido groups, with their numbers increasing as the pH rises. The coordination mode of gallium with thiouracil derivatives is also pH-dependent. At low pH values, the gallium ion coordinates primarily through the nitrogen atom, whereas at higher pH values, coordination occurs through both oxygen and sulfur donor atoms. Luminescence studies further confirm these changes in the internal coordination sphere of the gallium ion within the studied systems. The crystal structures of 6-methoxymethyl-2-thiouracil (MMTU) and the previously described 6-propyl-2-thiouracil (PTU) reveal the formation of strong hydrogen bond donors (NH groups) and acceptors (carbonyl oxygen and, potentially, the sulfur atom), which together promote the formation of infinite chains.

As for the combination of stability, the pH and coordination modes make thiouracil derivatives highly versatile ligands for gallium ions. These characteristics underscore their potential to create more effective and less toxic gallium-based therapies. Furthermore, the possible ability of these complexes to mimic iron ions makes them promising candidates for the treatment of conditions like cancer and bacterial infections.

This study paves the way for further exploration of gallium–thiouracil complexes, particularly in optimizing their properties for specific therapeutic applications. The findings contribute to the broader field of medicinal inorganic chemistry, providing a foundation for the development of innovative drugs with enhanced efficacy and reduced side effects and exploring novel coordination compounds with tailored properties to meet the growing demands of modern medicine.

## Figures and Tables

**Table 1:** The values of the overall protonation or stability constant (log *β*) and the equilibrium constants (log *K_e_*) for the examined systems.

Form	Log *β*	Reaction	Log *K_e_*
**TU**			
HL	6.95(6)	L + H^+^ ⇆ HL	6.95
GaLOH	3.34(4)	Ga^3+^ + L + H_2_O ⇆ GaL(OH) + H^+^	17.11
GaL(OH)_3_	−12.26(8)	GaL(OH) + 2H_2_O ⇆ GaL(OH)_3_ + H^+^	11.94
GaL_2_(OH)	9.53(8)	GaL(OH) + L ⇆ GaL_2_(OH)	6.19
GaL_2_(OH)_3_	−6.79(9)	GaL_2_(OH) + 2H_2_O ⇆ GaL_2_(OH)_2_ + H^+^	11.22
**MTU**			
HL	7.71(6)	L + H^+^ ⇆ HL	7.71
GaLOH	3.95(4)	Ga^3+^ + L + H_2_O ⇆ GaL(OH) + H^+^	17.81
GaL(OH)_2_	−3.13(8)	GaL(OH) + H_2_O ⇆ GaL(OH)_2_ + H^+^	6.76
GaL(OH)_3_	−11.64(7)	GaL(OH)_2_ + H_2_O ⇆ GaL(OH)_3_ + H^+^	5.35
GaL_2_(OH)	11.18(8)	GaL(OH) + L ⇆ GaL_2_(OH)	21.09
GaL_2_(OH)_2_	3.50(1)	GaL_2_(OH) + L ⇆ GaL_2_(OH)_2_ + H^+^	6.26
GaL_2_(OH)_3_	−5.00(1)	GaL_2_(OH)_2_ + H_2_O ⇆ GaL_2_(OH)_3_ + H^+^	5.19
GaL_2_(OH)_4_	−13.90(1)	GaL_2_(OH)_3_ + H_2_O ⇆ GaL_2_(OH)_4_ + H^+^	4.98
**PTU**			
HL	7.69(5)	L + H^+^ ⇆ HL	7.69
GaLOH	3.90(2)	Ga^3+^ + L + H_2_O ⇆ GaL(OH) + H^+^	16.68
GaL(OH)_2_	−3.54(6)	GaL(OH) + H_2_O ⇆ GaL(OH)_2_ + H^+^	6.32
GaL(OH)_3_	−14.19(9)	GaL(OH)_2_ + H_2_O ⇆ GaL(OH)_3_ + H^+^	3.12
GaL_2_(OH)_2_	1.76(9)	GaL(OH) + L + H_2_O ⇆ GaL_2_(OH)_2_ + H^+^	11.63
GaL_2_(OH)_3_	−6.47(7)	GaL_2_(OH)_2_ + H_2_O ⇆ GaL_2_(OH)_3_ + H^+^	5.52
**CTU**			
HL	4.01(7)	L + H^+^ ⇆ HL	4.01
GaL(OH)_2_	−8.35(5)	Ga + L + 2H_2_O ⇆ GaL(OH)_2_ + H^+^	19.19
GaL_2_(OH)_3_	−12.31(9)	GaL(OH)_2_ + L + 2H_2_O ⇆ GaL_2_(OH)_3_	23.58
**MMTU**			
HL	6.58(1)	L + H^+^ ⇆ HL	6.58
GaL(OH)	2.77(2)	Ga^3+^ + L + H_2_O ⇆ GaL(OH) + H^+^	2.77
GaL(OH)_2_	−4.06(5)	GaL(OH) + H_2_O ⇆ GaL(OH)_2_ + H^+^	6.93
GaL(OH)_3_	−11.66(4)	GaL(OH)_2_ + H_2_O ⇆ GaL(OH)_3_ + H^+^	6.16
GaL_2_(OH)	8.00(1)	GaL(OH) + L ⇆ GaL_2_(OH)	5.26

**Table 2:** The geometrical parameters of HBs.

D-H···A	D-H (Å)	H···A (Å)	D···A (Å)	D-H···A (°)	Ref.
**MTU**					
**N1-H1···S2i**	0.87 (2)	2.43 (2)	3.297 (2)	173.7 (19)	*
**N3-H3···O4ii**	0.88 (2)	1.92 (2)	2.808 (2)	177.3 (18)	*
**PTU**					
**N1A-H1A** **⋯** **S2Biii**	0.88	2.48	3.341 (2)	165	[29]
**N1B-H1B** **⋯** **S2Aiv**	0.88	2.50	3.361 (2)	167	[29]
**N3A-H3A** **⋯** **O4B**	0.88	1.94	2.797 (3)	163	[29]
**N3B-H3B** **⋯** **O4A**	0.88	1.98	2.826 (3)	161	[29]

Symmetry code(s): (i) -x, -y + 1, -z; (ii) -x + 1, -y + 2, -z + 1, (iii) x, y − 1, z; (iv) x, y + 1, z. * this work.

## Data Availability

Data are contained within the article.

## Supplementary Materials

The following supporting information can be downloaded at https://www.mdpi.com/article/10.3390/ijms252312869/s1.
