# Supplementary material for: Investigation of Gallium(III) Complexes with Thiouracil Derivatives: Effects of pH on Coordination and Stability
Source: Int J Mol Sci. 2024 Nov 29;25(23):12869. doi: 10.3390/ijms252312869 (PMC11641691; doi:10.3390/ijms252312869)
Supplement: Supplementary file 1 [file ijms-25-12869-s001.zip › ijms-3315283-supplementary.pdf]

**Supplementary Materials**

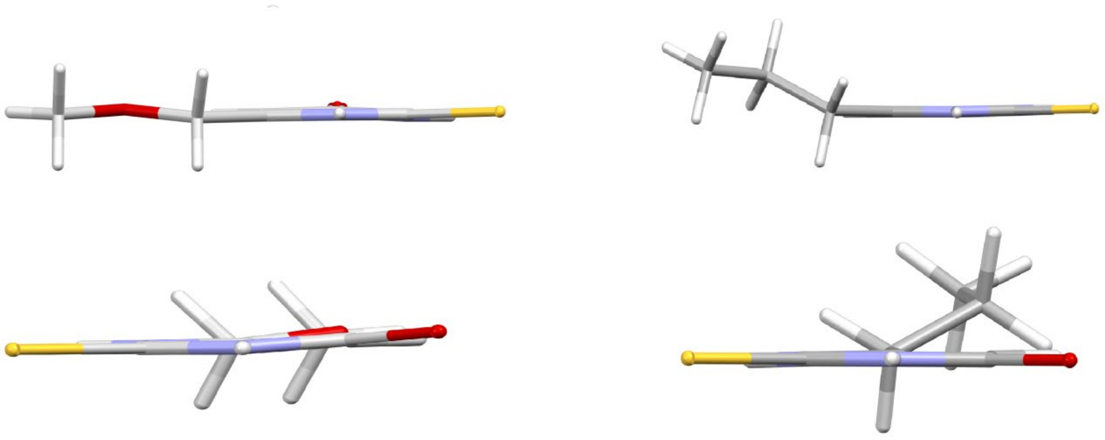

Figure S1 The view of MMTU (left) and PTU(right) from the aromatic ring  perspective

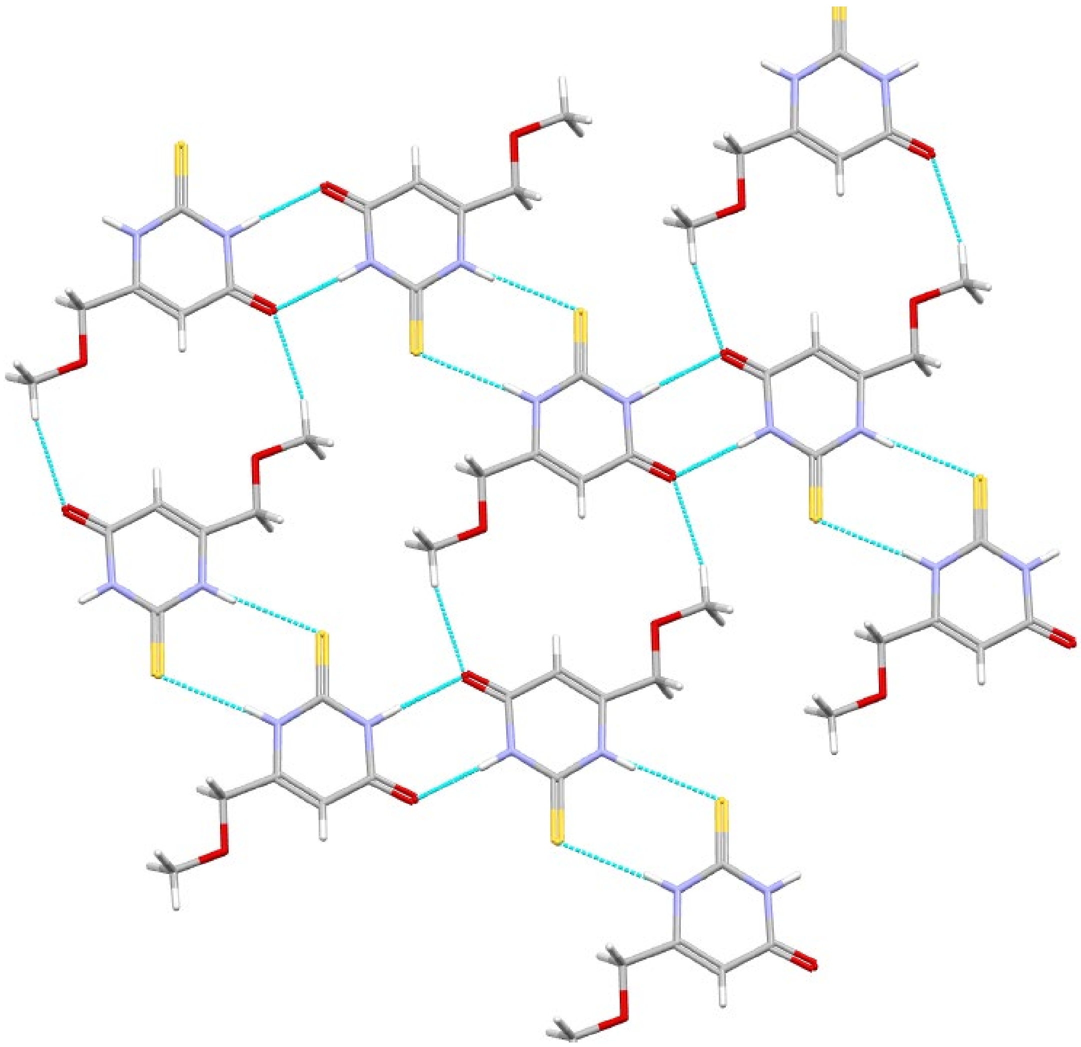

Figure S2 Two dimentional hydrogen bond layer in the crystal structure of MMTU

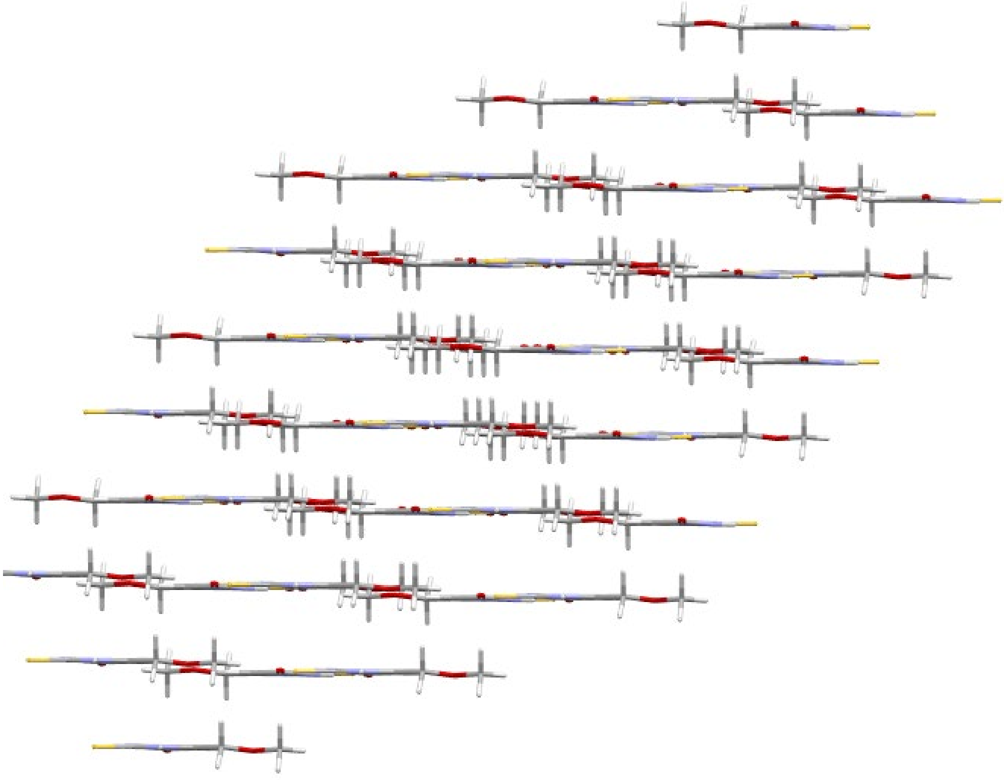

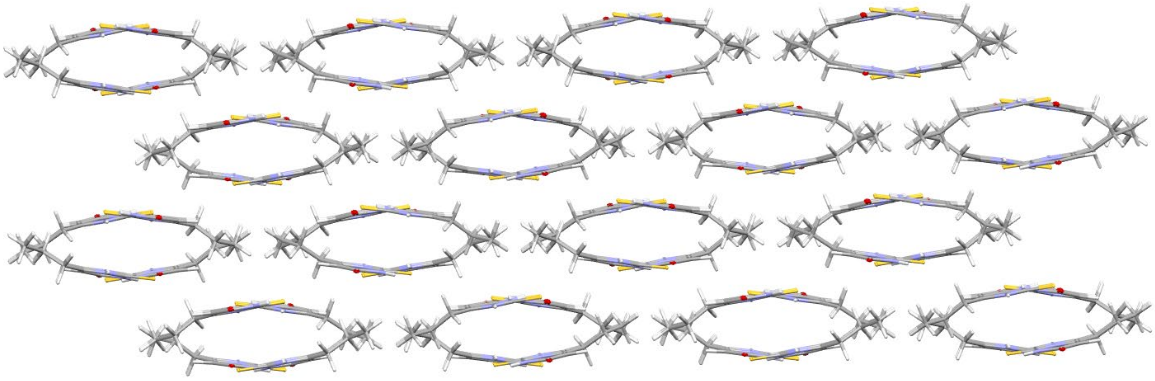

Figure S3 The crystal packing of MMTU (top) and PTU ( bottom) along the NH···S, NH···O hydrogen bonded chains

**Table S1.** Crystallographic data and refinement details.

| | **MMTU** |
|---|---|
| Crystal data | |
| Chemical formula | $C_6H_8N_2O_2S$ |
| $M_r$ | 172.20 |
| Crystal system, space group | Triclinic, $P\bar{1}$ |
| Temperature (K) | 130 |
| *a*, *b*, *c* (Å) | 4.4995 (1), 9.2328 (3), 9.9558 (3) |
| α, β, γ (°) | 112.581 (3), 91.546 (2), 97.313 (2) |
| $V$ (Å$^3$) | 377.48 (2) |
| $Z$ | 2 |
| Radiation type | Cu $K\alpha$ |
| μ (mm$^{-1}$) | 3.43 |
| Crystal size (mm) | 0.38 × 0.26 × 0.24 |
| Data collection | |
| Absorption correction | Multi-scan |
| $T_{min}$, $T_{max}$ | 0.492, 1.000 |
| No. of measured, independent and observed [$I > 2\sigma(I)$] reflections | 13348, 1526, 1507 |
| $R_{int}$ | 0.027 |
| (sin θ/λ)$_{max}$ (Å$^{-1}$) | 0.625 |
| Refinement | |
| $R[F^2 > 2\sigma(F^2)]$, $wR(F^2)$, $GooF$ | 0.031, 0.087, 1.07 |
| No. of reflections | 1526 |
| No. of parameters | 132 |
| $\Delta\rangle_{max}$, $\Delta\rangle_{min}$ (e Å$^{-3}$) | 0.35, -0.29 |
| CCDS deposition number | 2387155 |
